# A Novel Microfluidic Strategy for Efficient Exosome Separation via Thermally Oxidized Non-Uniform Deterministic Lateral Displacement (DLD) Arrays and Dielectrophoresis (DEP) Synergy

**DOI:** 10.3390/bios14040174

**Published:** 2024-04-04

**Authors:** Dayin Wang, Shijia Yang, Ning Wang, Han Guo, Shilun Feng, Yuan Luo, Jianlong Zhao

**Affiliations:** 1 State Key Laboratory of Transducer Technology, Shanghai Institute of Microsystem and Information Technology, Chinese Academy of Sciences, Shanghai 200050, China; dywang@mail.sim.ac.cn (D.W.);; 2 Center of Materials Science and Optoelectronics Engineering, University of Chinese Academy of Sciences, Beijing 100049, China; 3 School of Information Science and Technology, ShanghaiTech University, Shanghai 201210, China

**Keywords:** exosomes, deterministic lateral displacement (DLD), dielectrophoresis (DEP), microfluidic separation, thermal oxidation

## Abstract

Exosomes, with diameters ranging from 30 to 150 nm, are saucer-shaped extracellular vesicles (EVs) secreted by various type of human cells. They are present in virtually all bodily fluids. Owing to their abundant nucleic acid and protein content, exosomes have emerged as promising biomarkers for noninvasive molecular diagnostics. However, the need for exosome separation purification presents tremendous technical challenges due to their minuscule size. In recent years, microfluidic technology has garnered substantial interest as a promising alternative capable of excellent separation performance, reduced reagent consumption, and lower overall device and operation costs. In this context, we hereby propose a novel microfluidic strategy based on thermally oxidized deterministic lateral displacement (DLD) arrays with tapered shapes to enhance separation performance. We have achieved more than 90% purity in both polystyrene nanoparticle and exosome experiments. The use of thermal oxidation also significantly reduces fabrication complexity by avoiding the use of high-precision lithography. Furthermore, in a simulation model, we attempt to integrate the use of dielectrophoresis (DEP) to overcome the size-based nature of DLD and distinguish particles that are close in size but differ in biochemical compositions (e.g., lipoproteins, exomeres, retroviruses). We believe the proposed strategy heralds a versatile and innovative platform poised to enhance exosome analysis across a spectrum of biochemical applications.

## 1. Introduction

Exosomes, integral to intercellular communication, have attracted substantial interest across myriad biomedical research fields. These nano-sized extracellular vesicles are pivotal in mediating a myriad of physiological and pathological processes, including cancer progression, immune responses, and tissue regeneration [1,2,3,4]. These vesicles encapsulate a diverse array of biomolecules, including proteins, lipids, and nucleic acids, reflecting the molecular signature of the parent cell and thereby offering a snapshot of its health and activity [5,6,7,8]. However, the co-presence of several other types of EVs in biofluids often complicates the precise analysis of exosomes, underscoring the need for their separation from abundant sources like blood, urine, and saliva. Traditional exosome separation techniques, including ultracentrifugation, density gradient centrifugation, and methods relying on magnetic beads, are time-consuming and labor-intensive, while achieving exosome separation of low purity [9,10,11].

In recent years, the adaptation of microfluidics to separate and capture exosomes has gradually grown in prominence. A series of technological solutions have been developed for manipulating the fluid flow. Nanoporous membranes integrated into microfluidic chips intercept particles larger than the pore size while smaller components pass through [12]. A nanowire-based strategy achieves exosome trapping through spatial hindrance [13]. The viscoelastic flow method achieves particle sorting based on the elastic lift forces exerted on particles in a viscoelastic medium [14,15,16]. Deterministic lateral displacement (DLD), a label-free method, enables the sorting and enrichment of nanoparticles based on size [17,18,19,20,21]. Dielectrophoresis (DEP) separates particles based on their intrinsic dielectric properties rather than size [22,23]. Among these methods, DLD provides a continuous process that circumvents the drawbacks of nanoporous membranes, nanowires, and viscoelastic methods by avoiding blockages and the introduction of impurities. The high separation resolution of DLD enables it to stand out among various microfluidic separation techniques [24]. DEP proves more effective for micrometer-scale particles but shows limited efficacy with nanometer-scale particles.

**Figure 1:**
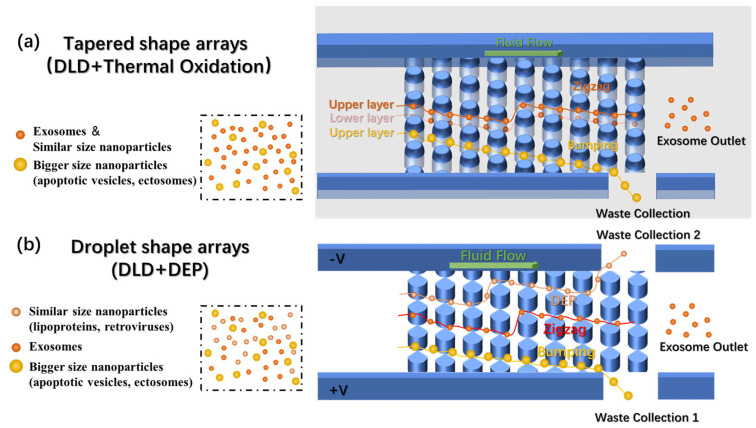
The overall design of the chips. (**a**) Schematic of the tapered shape DLD arrays fabricated after thermal oxidation showing how exosomes and larger nanoparticles flow through. (**b**) Schematic of the droplet-shaped arrays combining DEP with DLD arrays, showing how exosomes, bigger nanoparticles, and similarly sized nanoparticles flow through.

In this work, we propose a novel approach via thermally oxidized DLD arrays with tapered shapes to improve the separation performance of nano-sized particles. First, by applying thermal oxidation to the column array fabricated through micrometer-scale lithography and etching, nanometer-level inter-column spacing is achieved, which significantly reduces fabrication costs by avoiding the use of high-precision lithography. Second, while previous studies have also applied thermal oxidation in DLD technology [25], we critically realized that thermal oxidation could transform vertical columns into tapered shapes, causing the separation threshold of the DLD array to deviate from its intended design. We attempt to elucidate this phenomenon with further experimental investigation. The tapered structure (Figure 1a) after thermal oxidation employs the wider upper layer to exert the DLD effect on larger impurity particles, while both the upper and lower layers allow exosomes to pass through unimpeded. We used mixtures of polystyrene nanoparticles and exosomes to verify the separation performance of the device, resulting in >90% purity from collected samples. In addition, we aim for a design capable of separating extracellular vesicles (EVs) of similar sizes (e.g., lipoproteins, retroviruses, and exoparticles) that combines dielectrophoresis (DEP) and DLD. By designing a droplet-shaped structure to replace the traditional cylindrical structure of DLD (Figure 1b), we tested the feasibility of such a design through a simulation study. The overall strategy in our work demonstrated significant promise as a multifunctional and label-free methodology for exosome separation, offering enhanced specificity in particle discrimination through the integration of size-based and electrical-property-based separation mechanisms.

## 2. Materials and Methods

### 2.1. Theory

#### 2.1.1. DLD

**Figure 2:**
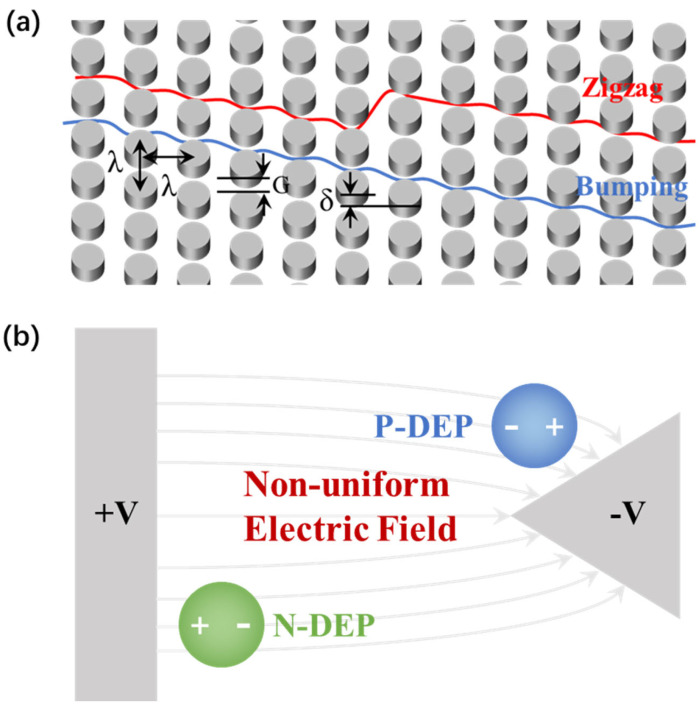
(**a**) Schematic of the DLD arrays that illustrates the array parameters of the pillar gap size, *G*, pillar pitch, *λ*, and row-to-row shift, *δ*, and the two different modes separate particles flow in. (**b**) Schematic of dielectrophoresis, showing how n-DEP particles and p-DEP particles act in a non-uniform electric field.

DLD represents a size-based particle separation methodology and exhibits significant potential for the continuous, label-free separation of particles that are smaller than the microfluidic array’s feature size. A DLD device incorporates a particle separation region, which consists of an array composed of microposts or columns crafted from a rigid material [26], as depicted in Figure 2a. The width of the gaps, perpendicular to the direction of fluid flow, is denoted as *G*, and the center-to-center spacing between the posts is represented by *λ*. The lateral displacement between adjacent rows of posts is referred to as *δ*. The crucial parameter in the design of a DLD device is the shift fraction (*ε*), which is defined as

*ε* = *δ*/*λ*, (1)

The operational principle of the DLD device hinges on a critical size threshold (*D_C_*). Particles exceeding this threshold size are laterally displaced and follow bumping mode, a trajectory along the ‘blue line’ of the array, whereas particles below this threshold size flow in zigzag mode, the red line, with no displacement occurring. However, before establishing the calculation model for determining *D_C_*, we first need to conduct a fluid dynamics analysis of the fluid within the device. Below, we will introduce the Navier–Stokes equations and the Reynolds Number.

A large number of phenomena for fluid can be described by the Navier–Stokes equation [26]:

(2) $$\rho \frac{\partial \vec{\nu }}{\partial t}+\rho \left(\vec{\nu }\cdot \nabla \right)\vec{\nu }=-\nabla p+\mu \nabla^{2}\vec{\nu }$$

where ρ is the density of the fluid, $\vec{\nu }$ is the velocity vector, *p* is the pressure, and *μ* is viscosity. The Reynolds Number (*Re*) serves as a measure that contrasts inertial forces against viscous forces, thereby quantifying the respective significance of these forces in influencing fluid flow. It is defined as [26]:

(3) $$Re=\frac{\rho \nu L}{\mu }$$

where *v* is the speed of the fluid (0.25 mm/s in our experiment) and *L* (approximately 1 mm in our design) is a characteristic length. The ρ and *μ* for 1xPBS used in our experiment are close to those for water (10^3^ kg/m^3^ and 10^−3^Pa·s). The calculated *Re* in our experiment is 0.25 < 1, which means that viscous damping rapidly dissipates kinetic energy (both translational and rotational) from a fluid element, rendering inertial forces negligible and thus permitting their exclusion from the Navier–Stokes equation [24]. The modified Stokes’ Equation (2) is

(4) $$\rho \frac{\partial \vec{\nu }}{\partial t}=-\nabla p+\mu \nabla^{2}\vec{\nu }$$

which reveals a laminar flow. In this scenario, the optimal model for the critical threshold (*D_C_*) of DLD device is defined as [26]

*D_C_* = 1.4*Gε*^0.48^ (5)

#### 2.1.2. DEP

Dielectrophoresis (DEP) describes the displacement of a particle within a non-uniform electric field [27], as depicted in Figure 2b. When the particle is in an electric field, an induced charge appears on the particle’s surface, engendering a polarization aligned with the electric field. In uniform electric fields, the polarized charges experience a homogeneous force, leading to no particle displacement, unless the particle inherently possesses a net charge. However, in a non-uniform electric field, the forces diverge, thereby leading to the particle’s displacement. 

The direction of the DEP force depends on the comparative electrical polarizability of the particle relative to its suspending medium. When the particle’s polarizability surpasses that of the medium, the DEP force aligns with the electric field gradient, which is called positive dielectrophoresis, p-DEP. On the contrary, if the electrical polarizability of the particle is lower than that of the medium, the DEP force and the displacement vector oppose the field gradient, denoting negative dielectrophoresis, n-DEP. The composition, morphology, and phenotype of the particles in conjunction with electric field frequency significantly influence its polarizability. The DEP force on a spherical particle is given by [28]

*F*_*DEP*_ = 2*πε*_*m*_*r*^3^*Re*[*f*_*CM*_] · ∇|*E_rms_*|^2^ (6)

where *r* is the radius of particles; *ε_m_* is the permittivity of the medium; *f_CM_* is the Clausius–Mossoti factor; and *E_rms_* is the root mean square value of an electric field. *Re*[*f_CM_*] means a real part of the *f_CM_*, which can be represented as follows [28]:

(7) $$\;\;\;f_{CM}=\frac{\varepsilon_{p}^{*}-\varepsilon_{m}^{*}}{\varepsilon_{p}^{*}+2\varepsilon_{m}^{*}}$$

where ε* is the complex permittivity (ε* = *ε* − *jσ*/*ω*); *σ* is the conductivity; and *ω* is the electric field frequency. Subscripts *p* and *m* mean the particles and the medium, respectively. *Re*[*f_CM_*] > 0 means that particles show p-DEP response (the blue particle), while *Re*[*f_CM_*] < 0 means n-DEP response (the green particle).

For the analysis of *f_CM_* on cells, the spherical single-shell model is commonly utilized [29,30]. This model treats the particles as homogenous spherical bodies while segmenting them into two regions: the shell and the interior. It utilizes aggregated dielectric parameters from these regions along with the suspending medium to predict the DEP response as a function of frequency [31]. Exosomes, possessing a single-layer membrane structure similar to cells, have more uniformly distributed contents [1,2,3,4,5,6]; thus, the single-shell model will better reflect their electrical properties [29,32]. Below, we will derive the function describing the relationship between the *f_CM_* and the applied electric field frequency through a spherical single-shell model.

According to the model, the effective complex permittivity of exosomes $\varepsilon_{ex}^{*}$ can be described as:

(8) $$\varepsilon_{ex}^{*}=\frac{c_{mem}^{*}r\varepsilon_{c}^{*}}{c_{mem}^{*}r+\varepsilon_{c}^{*}}\;$$

where $\varepsilon_{c}^{*}$ is the complex permittivity of the contents of exosomes and $c_{mem}^{*}$ is the complex capacitance of exosome membrane, which can be defined as:

(9) $$\;c_{mem}^{*}=c_{mem}+g_{mem}/j\omega$$

where $c_{mem}$ and $g_{mem}$ are the capacitance and conductance (often negligible) of the exosome membrane per unit area. Thus, the analysis of *f_CM_* is defined as:

(10) $$f_{CM}\left(\omega \right)=-\frac{\omega^{2}\left(\tau_{m}\tau_{c}^{*}-\tau_{c}\tau_{m}^{*}\right)+j\omega \left(\tau_{m}^{*}-\tau_{c}^{*}-\tau_{m}\right)-1}{\omega^{2}\left(2\tau_{m}\tau_{c}^{*}+\tau_{c}\tau_{m}^{*}\right)-j\omega \left(\tau_{m}^{*}+\tau_{c}^{*}+2\tau_{m}\right)-2}$$

where the time constants are defined as follows: $\tau_{c}=\frac{\varepsilon_{c}}{\sigma_{c}}$, $\tau_{c}^{*}=\frac{c_{c}^{*}r}{\sigma_{c}}$, $\tau_{m}=\frac{\varepsilon_{m}}{\sigma_{m}}$ and $\tau_{m}^{*}=\frac{c_{m}^{*}r}{\sigma_{m}}$.

### 2.2. DLD Array Fabrication Process

**Figure 3:**
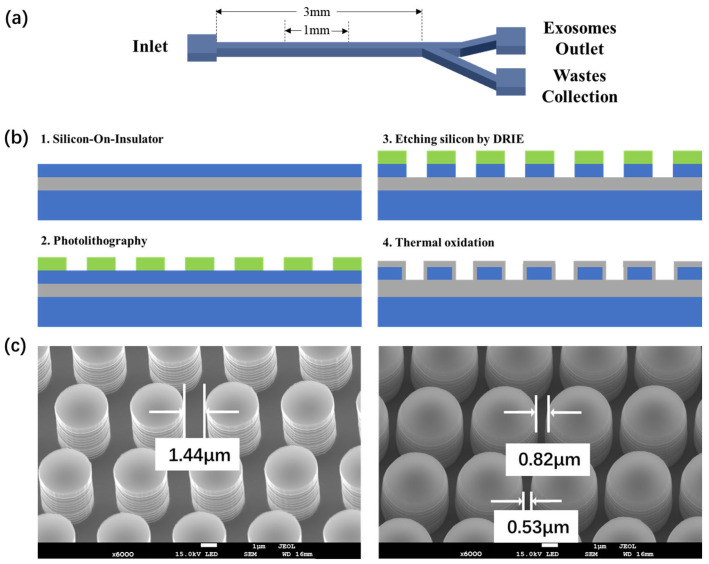
(**a**) Schematic of the DLD exosome separation chip. (**b**) The fabrication process flow of the non-uniform DLD arrays inviting thermal oxidation. (**c**) The SEM images of the perpendicular arrays fabricated through micron-scale lithography etching (**left**) and the tapered arrays shaped by thermal oxidation (**right**).

The DLD array fabrication process is shown in Figure 3b. The silicon-on-insulator wafer was used to isolate the sidewall and the arrays inside after etching up the whole top silicon layer. The parameters are shown in Table S1 (in Supplementary Material). To enhance the adhesion of the photoresist to the silicon wafer, we first applied a spin coating of HMDS (Hexamethyldisilazane) adhesion promoter, using coating parameters of 2000 rpm for 20 s. The spin-coating parameters for the S1805 photoresist were set at 3000 rpm for 30 s. The photoresist is pre-baked at 110 °C for 90 s. The photolithography system (SUSS Mask Aligner MA6, SUSS MicroTec, Garching, Germany) is set to Vacuum mode, with an exposure time of 3 s. Development is conducted in ZX-238 Positive Photoresist Developer for approximately 40 s, completing the photolithography process for the pattern. Utilizing the STS etching machine, the device undergoes a deep reactive ion etching (DRIE) process to a depth of 2 µm. The etching parameters are detailed in Table S2 (in Supplementary Material). The thermal oxidation is conducted according to the standard 1.5 µm thermal oxidation protocol.

### 2.3. Soft Lithography and PDMS Preparation

Given the relatively shallow etching depth of the chips used in this study, to prevent collapse and subsequent blockage of the inlet and outlet areas with low aspect ratios during bonding with the PDMS layer, we employed soft lithography to fabricate molds. This process allowed for the creation of PDMS with upward-facing grooves corresponding to the regions of the inlets and outlets, ensuring their structural integrity and preventing occlusion.

The mold fabrication was carried out on standard silicon substrates. We utilized SU8-3025 photoresist for the creation of patterns. First, we dispensed 1 mL of photoresist for each inch (25 mm) of substrate diameter. Then, we spun at 500 rpm for 5–10 s with acceleration of 100 rpm/s. After that, we spun at 3000 rpm for 30 s with acceleration of 300 rpm/second. We soft baked for 10 min at 95 °C. We exposed at 200 mJ/cm^2^. We post-baked for 5 min at 95 °C. Finally, we developed for 8 min in PGMEA solution. To ensure repeated use of the mold, a layer of HMDS (Hexamethyldisilazane) was sputtered onto the mold to prevent the impact on the patterns during PDMS demolding.

PDMS polymer was placed in a vacuum pump to remove internal bubbles. Subsequently, the degassed PDMS was poured onto the mold and cured on a hot plate at 110 °C for approximately 2 h before being peeled off. The chips were cleaned using acetone and isopropanol. The PDMS slabs and the chips were then treated with oxygen plasma and bonded with each other. The assembled device was placed into an oven at 60 °C for 3 h to enhance bonding. The soft lithography and PDMS preparation process flow are shown in Figure S1 (in Supplementary Material).

### 2.4. Particle Sample Preparation

In our work, we utilized standard polystyrene fluorescent particles of 600 nm and 100 nm (Thermo Fisher Scientific, Waltham, MA, USA). The 600 nm particles were labeled with Fluoro-Max Blue dye (ex/em: 365/447, 412/473 nm), while the 100 nm particles were marked with Fluoro-Max Red dye (ex/em: 542/612 nm). Solutions of 600 nm and 100 nm standard polystyrene fluorescent particles, with identical solid contents, were combined at a volume ratio of 200:1, equating to a quantity ratio of approximately 1:1.

### 2.5. DLD Experimental Instruments

Sample mixture fluid was injected into the microfluidic chips using a syringe pump (LSP02-2A, LongerPump, Baoding, China) at various constant flow rates. The fluorescent images were obtained using a biological microscope (BX53(LED), Evident, Tokyo, Japan). We employed the DAPI (ex/em: 365/473 nm) and TRITC (ex/em: 543/585 nm) excitation groups.

### 2.6. EV Sample Preparation

EVs were isolated from the cell culture medium of HaCaT cells. These cells were cultured in high-glucose Dulbecco’s Modified Eagle’s Medium (DMEM) enriched with 10% exosome-depleted fetal bovine serum (Wisent, Toronto, ON, Canada) within a 5% CO_2_, 37 °C incubator (Thermo Scientific, USA). After cultivating for 48 h to achieve approximately 80% cell confluence, the medium was replaced with a serum-free variant, and the supernatant, rich in EVs, was collected after an additional 24–48 h. The isolation of EVs was performed using differential centrifugation at 4 °C. The process began with the initial centrifugation of the collected supernatant (300 mL) at 500× *g* for 5 min, followed by 2000× *g* for 30 min to eliminate cells, and then at 10,000× *g* for 60 min. Subsequently, the supernatant was transferred into ultracentrifuge tubes and spun at 120,000× *g* for 70 min at 4 °C. Post-centrifugation, the supernatant was carefully decanted, and the pellet was reconstituted in sterile 1X PBS. This resuspension was then collected to procure exosomes.

### 2.7. Nanoparticle Tracking Analysis (NTA)

To assess the recovery rate and purity of the isolated 100 nm polystyrene nanoparticles and exosomes, separately, Nanoparticle Tracking Analysis (NTA; NanoSight NS300, Malvern Instruments, Malvern, UK) was employed to measure the size distributions of both the initial and the processed samples. All samples were diluted in 1xPBS to concentrations ranging from 2 × 10^8^ to 1 × 10^9^ particles/mL to ensure optimal accuracy. The acquisition of size distribution data was performed using the NTA 3.2 Analytical Software Suite (27 November 2015, NanoSight NTA software update v3.2 | Malvern Panalytical). These measurements were executed at a controlled temperature of 20 °C.

### 2.8. Scanning Electron Microscope (SEM)

After etching, the four-inch wafers were sectioned into individual chips for observation with a Supra 55 (Zeiss, Jena, Germany) scanning electron microscope (SEM). The silicon on the chip exhibited excellent conductivity, allowing for observations to be conducted at room temperature (298 K) with a 30-degree tilt in a vacuum. After undergoing thermal oxidation, the surface of the chips was coated with an insulating layer of silicon dioxide. For enhanced visualization of the column sidewalls, it is advisable to sputter-coat a thin layer of gold to improve conductivity prior to SEM observation. The chips after thermal oxidation in this work did not undergo a gold sputtering process.

## 3. Results

### 3.1. DLD Chip Design and Fabrication

Figure 3a first shows the schematic of the DLD exosome separation chip. The chip consists of one inlet, a straight DLD array separation region, and two outlets (one for exosome collection, the other for waste). The whole length of the microfluidic channel is 3 mm. The length of the DLD separation area is set at 1 mm, which is determined based on the requirements for the Re number, as outlined in Equation (2). With a fluid flow rate of 0.25 mm/sec within our DLD device, maintaining the fluid as laminar flow necessitates that the length of the sorting area does not exceed 4 mm. A length of 1 mm provides sufficient margin to vary the flow rate. The width of the device is set at 90 μm. This specification arises from our designed shift fraction of 0.1, and, in order to ensure the array sufficiently separates all particles completely, the width must be less than 1000 μm length multiplied by the shift fraction of 0.1, equating to less than 100 μm. The depth of the microchannel is controlled at 2 µm through a DRIE etching process, which was identified as the optimal array depth for thermal oxidation effects. In our experimental design, we first employed standard fluorescent polystyrene nanoparticles with diameters of 100 nm and 600 nm as proxies for exosomes and larger nanoparticles present in actual bodily fluids, respectively. We therefore set the DLD separation threshold at 300 nm. According to Equation (2), we need a column-to-column spacing of ~800 nm to achieve the threshold of 300 nm with a displacement fraction of 0.1 in order to realize DLD separation between the two selected sizes.

Typically, the nano-scale linewidth necessitates the use of high-precision fabrication equipment, such as stepper lithography systems, thereby significantly increasing device production costs. To address this issue, we incorporated thermal oxidation into the array construction process. The left image of Figure 3c displays the micrometer-scale array produced through micrometer-level lithography and etching. After thermal oxidation, as shown in the right image of Figure 3c, the spacing between arrays was reduced to the nanometer scale, meeting the requirements of DLD devices for the separation of nanometer-scale particles. It is worth noting that during the thermal oxidation of the device, only the upper ends and the sides of the pillars are exposed to oxygen species. This leads to the silicon at the lower part of the pillars undergoing lateral thermal oxidation growth exclusively, whereas the silicon at the upper ends experiences both lateral and vertical thermal oxidation growth during thermal oxidation (Figure S2 in Supplementary Material). Consequently, the lateral thermal oxidation growth at the upper ends is less extensive than at the lower ends, resulting in the formation of a tapered structure. According to Equation (2) from the DLD theory section, such differential spacing between the upper and lower layers leads to distinct critical diameters (*D_C_*) for each; as such, the arrays as a whole no longer maintain a precise separation threshold. To minimize the impact of this tapered structure on exosome separation, we tried to refine the thermal oxidation process by precisely controlling the spacing between the upper and lower layers. We conducted the same thermal oxidation process on columns with different etching depths and found that in columns of smaller depth, the non-uniformity of the upper and lower layer was less pronounced. A possible explanation is that when the columns are of smaller depth, the volume of silicon at the upper layer is smaller, leading to a reduced amount of silicon being oxidized both laterally and vertically. Moreover, oxygen can distribute more evenly among columns of smaller depth, which is beneficial for the uniformity of the overall thermal oxidation. At an etching depth of 2 µm, we achieved a relatively optimal thermal oxidation effect. The upper layer, as depicted in Figure 3c, initially spanning 1.44 µm, undergoes reduction to 0.82 µm, leading a 300 nm threshold, thereby enabling the separation of most impurity particles without exerting the DLD effect on exosomes. In contrast, the reduction of the lower layer from 1.44 µm to 0.53 µm establishes a separation threshold of 200 nm, ensuring that exosomes (below 200 nm) remain unaffected by the DLD phenomenon. It is worthwhile to stress that the utilization of thermal oxidation in device fabrication unequivocally results in the formation of such tapered structures. Previous work that also employs thermal oxidation to create nano-sized DLD pillars [25] did not recognize that such a variation in gap sizes in the vertical direction plays an important role in explaining the actual DLD behaviors of the particles. Here, we pay specific attention to this phenomenon when designing the pillar sizes and oxidation parameters. Furthermore, such a process markedly reduces the costs associated with chip fabrication by circumventing the need for high-precision lithography techniques.

### 3.2. DLD Separation Performance

**Figure 4:**
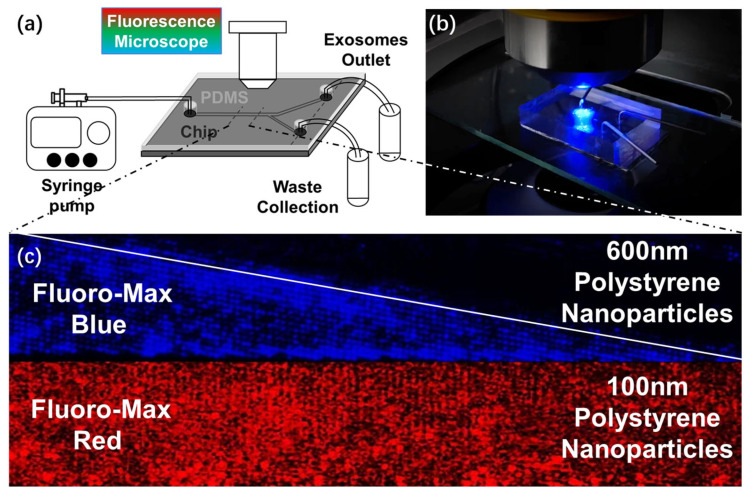
(**a**) Schematic of the microfluidic DLD experiment setup. (**b**) Actual image of the chip used for the DLD experiment. (**c**) Image obtained through fluorescence microscopy reveals distinct behaviors: 600 nm polystyrene nanoparticles, labeled with Fluoro-Max blue dye, exhibit DLD phenomena, whereas 100 nm polystyrene nanoparticles, marked with Fluoro-Max red dye, demonstrate no displacement.

Figure 4a,b depict the microfluidic experimental setup and the actual image of the device. A syringe pump injects sample solutions into the chip at a constant flow rate. The chip is enclosed by a PDMS layer, whose transparency allows for the observation of particle trajectories via fluorescence microscopy. During device operation, the larger nanoparticles were heavily influenced by the DLD effect, resulting in their deviation along the streamline and subsequent collection from the waste outlet. On the contrary, the smaller-sized exosomes flow directly towards the exosome outlet.

For the experiment, a mixture of 600 nm (labeled with Fluoro-Max blue dye) and 100 nm (labeled with Fluoro-Max red dye) standard fluorescent polystyrene nanoparticles was injected into the chip at a flow rate of 5 × 10^−2^ μL/min. Within the solution, we introduced a small quantity of Tween-20 surfactant to mitigate the adhesion of the fluorescent polystyrene nanoparticles to the PDMS layer, thereby preventing any interference with the microscopic observations. As shown in Figure 4c, during a typical device run, the streamlines of 600 nm polystyrene nanoparticles form a triangular pattern, indicating the displacement of these larger nanoparticles due to DLD, whereas the 100 nm nanoparticles pass through unaltered. We also present a dynamic video showcasing the movement of particles in Video S1.

**Figure 5:**
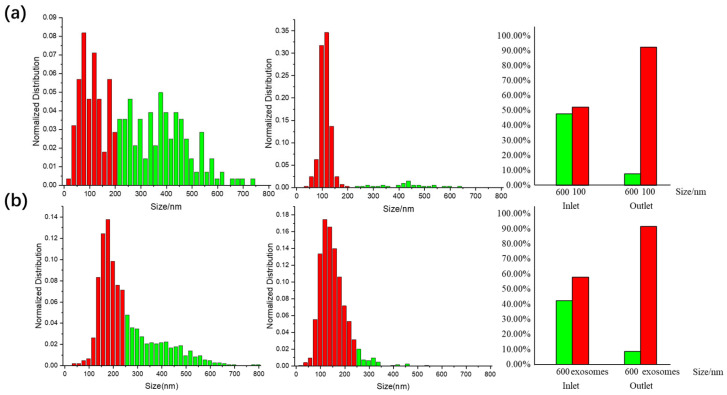
NTA results and the purity behavior of the two experiments. (**a**) Normalized distribution of the mixture sample of 100 and 600 nm particles before and after separation in NTA test, (**b**) Normalized distribution of the mixture sample of HaCaT cell exosomes and 600 nm particles before and after separation in NTA test.

Subsequent collection and analysis of the sample solutions from both the inlet and the exosome outlet through Nanoparticle Tracking Analysis (NTA), as demonstrated in Figure 5a, confirm that the 100 nm nanoparticles have been effectively separated from the 600 nm ones, resulting in a purity from 52.31% to 92.31% from the outlet collection. Next, we further examined the separation performance with the mixture of HaCaT cell exosomes and 600 nm nanoparticles to evaluate the chip’s efficacy in a real bodily fluid context. The same experimental parameters as those used in the fluorescent polystyrene nanoparticle experiment were maintained. Our device demonstrated a significant reduction of 600 nm polystyrene particles and larger extracellular nanoparticles in the exosome sample post-separation. The purity of exosomes collected at the outlet was measured at 91.47% via NTA, a significant increase compared to 57.84% in the original sample, as shown in Figure 5b, indicating the chip’s robust capability of separating exosomes from complex biological mixtures.

### 3.3. Simulation of the Synergistic Effect of DLD and DEP

**Figure 6:**
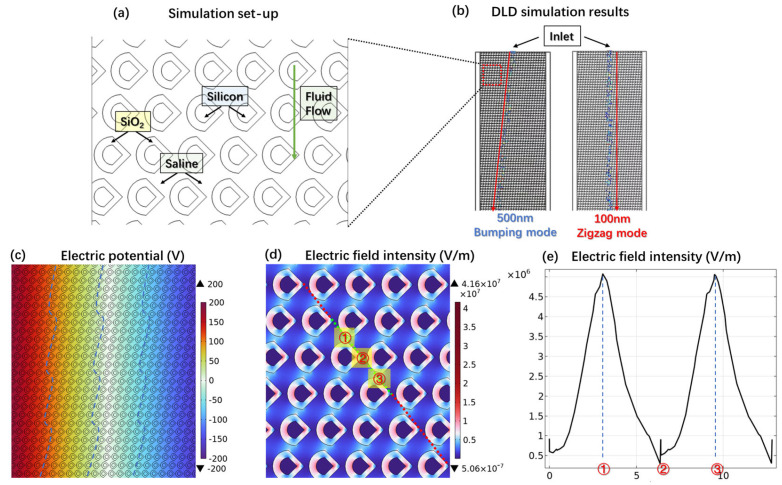
(**a**) Structure and components of the droplet-shaped columns synergistically employing deterministic lateral displacement and dieletrophoresis in simulation. (**b**) Particle-tracking simulation results of 500 nm polystyrene particles on the left and 100 nm polystyrene particles on the right. (**c**) Electric potential of the whole fluid channel and the wavy pattern longitudinal equipotential lines. (**d**) Electric field intensity distribution. (**e**) The line graph of the electric field intensity values along the green line in (**d**).

Famously, DLD faces challenges in the separation of extracellular nanoparticles of similar sizes [14], while the DEP effect is less potent at the nano scale compared to the separation of micron-scaled particles [22]. In pursuit of enhancing the precision of separating nanoparticles of similar sizes yet distinct biochemical compositions, such as lipoproteins, exomeres, and retroviruses, we propose here a strategy to integrate these two microscopic effects. As illustrated in Figure 6a, the key point of our design is an array of nano-scale, droplet-shaped pillars. This configuration not only preserves the characteristic layout of DLD arrays but also facilitates the generation of non-uniform electric fields, a crucial factor for implementing DEP.

By applying electrical excitation across the flow channel’s boundaries in the left and right sides, the droplet-shaped pillar arrays within the microfluidic region generate highly non-uniform electric fields that are overall perpendicular to the direction of the fluid flow. Such electric fields enhance the device’s overall ability to separate exosomes (ranging from 30 to 150 nm) from particles of similar sizes, such as lipoproteins (5 to 200 nm) and retroviruses (80 to 150 nm). Despite their size similarities, these nanoparticles exhibit significant variance in their biochemical compositions and structures [33], resulting in disparate electrical properties, as indicated in Equations (6)–(10).

By changing the frequency of the applied electrical signal, it is feasible to manipulate the complex permittivity of exosomes, similarly sized nanoparticles, and the surrounding medium. Our strategy is to align the complex permittivity of exosomes to zero, whereas that of similar nanoparticles deviates from zero. Under such conditions, exosomes are exempt from the influence of DEP forces, while other nanoparticles are experiencing effective DEP forces. This differential application of DEP forces enables the refined separation of exosomes from particles with overlapping size ranges but divergent biochemical identities. In determining the frequency, we plotted the real part of the Clausius–Mossotti factor (*f_CM_*) as a function of the frequency of exosomes and BSA (similar to lipoprotein, as an impurity) based on Equation (10), as shown in Figure S3a (in Supplementary Material). We found that within the 100–1000 kHz frequency range, the *Re*[*f_CM_*] for exosome is close to 0, while for BSA it is 1. This frequency range is well-suited to our needs. Furthermore, we noticed that the *Re*[*f_CM_*] function plots for gold nanoparticles and polystyrene nanoparticles closely resemble those for exosome and BSA, shown in Figure S3b (in Supplementary Material). This suggests that we could use gold and polystyrene nanoparticles for preliminary non-biological sample experiments in future studies.

We initially employed a particle tracking simulation to investigate whether this novel droplet-shaped structure possesses DLD functionality. The simulation setup is illustrated in Figure 6a, where the spaces between the shapes are filled with saline and the fluid characteristic is a laminar flow at a velocity of 300 µm/s. The gap between the droplet-shaped structures is set to 800 nm, with a shift fraction of 0.1. Particles 500 nm and 100 nm in diameter were injected into the middle of the microfluidic channel entrance at intervals of every 0.01 s for a duration of 24 s. The simulation results, shown in Figure 6b, clearly demonstrate that the 500 nm particles exhibit obvious lateral displacement, aligning with the DLD bumping mode, while the trajectory of the 100 nm particles is consistent with the zigzag mode. This indicates that our droplet-shaped array retains the functionality of DLD.

We further verified the DEP performance of this droplet-shaped structure. Our approach integrates the thermally oxidized, non-uniform DLD arrays with DEP functionality by configuring the individual pillar structure into two distinct layers, as Figure 6a shows. The core layer is composed of silicon, whereas the exterior layer is fabricated from SiO_2_. The space within the structure is defined as saline solution to simulate the real bodily fluid. A voltage of 400 Vpp at a frequency of 100 kHz was applied across the microfluidic flow channel. Figure 6c displays the two-dimensional electric potential distribution within the flow channel, revealing that the longitudinal equipotential lines exhibit a wavy pattern closely resembling the inclined arrangement of columns within the channel. The significantly lower relative dielectric constant of SiO_2_ (3.9) compared to saline solution (80) and silicon (11.7) results in the highest electric field strength being generated within the silicon dioxide layer, as depicted by the two-dimensional electric field distribution in Figure 6d. Consequently, the voltage drop is primarily concentrated within the silicon dioxide layer. The inclined arrangement of the SiO_2_ pillars alters the uniform field strength distribution typically observed between parallel plate capacitors, leading to the formation of such wavy equipotential lines. Figure 6e displays the electric field distribution along the green diagonal line in Figure 6d. As is evident from the figure, a periodic electric field with a substantial gradient is generated along the diagonal. This phenomenon is precisely facilitated by the sharp tip of the droplet-shaped design we have implemented. The electric field lines densely converge at the sharp point marked as position 1, creating a region of high electric field intensity. In contrast, the transformation of the original circular shape into a droplet shape significantly enlarges the space above and below position 2, causing the longitudinal distribution of electric field lines to become sparse, thereby resulting in a region of lower electric field intensity. Consider a particle experiencing a positive DEP force. When positioned at location 1, a particle proceeds to travel with the fluid flow, gliding along the inclined sidewall toward location 2. Upon reaching location 2, the particle is drawn towards regions of higher electric field intensity, prompting its displacement to location 3. As this cyclic process persists, the particle is progressively displaced towards the right, a movement mainly governed by the DEP effect.

## 4. Discussion

In this study, we propose a novel strategy employing thermally oxidized non-uniform DLD arrays with tapered shapes to improve the separation performance of nano-sized particles. Although thermal oxidation has been utilized in the fabrication process of DLD arrays in previous work, the impact of the resultant tapered structures has often been overlooked. This oversight led to discrepancies between the device’s real separation threshold and the expected one [25], thus limiting the separation efficiency of the device. In our work, we precisely control the linewidth of the upper and lower layers on the same DLD pillars, thus enabling the upper layer to apply the DLD effect on impurity particles while ensuring that neither layer affects exosomal small particles. In experiments involving fluorescent nanoparticles and biological samples with exosomes, we achieved purities for target particles exceeding 90%. This technique preserves the benefits of conventional DLD methods, being label-free, labor-efficient, and field-free, while minimizing damage to exosomes. Additionally, it circumvents the need for high-precision lithography, thereby substantially lowering the fabrication costs associated with traditional DLD approaches. Furthermore, we propose a synergistic approach combining DEP and DLD to overcome the intrinsic size-based sorting limitation of DLD, which leads to its inability to separate exosomes from nanoparticles of similar sizes (e.g., lipoproteins, retroviruses, and exoparticles). This design not only retains the functional characteristics of DLD but also helps to create a highly non-uniform electric field between the columns after applying alternating voltage on both sides of the array, thereby generating strong DEP forces. By modulating the frequency of the alternating voltage, it is possible to adjust the DEP effects on particles with diameters similar to exosomes to a direction opposite to that of DLD while not exerting force on the exosomes themselves. Thus, the device is capable of separating exosomes based on particle size through DLD and further distinguishing impurities with dimensions similar to exosomes based on the particles’ intrinsic electrical properties through DEP. While our device demonstrates potential, there is room for improvement. The throughput and the fluid flow rate of our DLD device are at a low level, leading to long separation times for samples. In future iterations, we plan to implement multi-channel parallel injection and widen and deepen the flow channels to increase the overall device throughput. We have realized the fabrication of the critical pointed structures of the droplet-shaped arrays essential for the effective implementation of the DEP effect. We will soon validate the simulation results of our DEP design through experimental verification, using the outcomes to further refine our device design.

## 5. Conclusions

In summary, we demonstrated a novel microfluidic exosome separation device featuring thermally oxidized non-uniform deterministic lateral displacement (DLD) arrays. By precisely adjusting the linewidth of the upper and lower layers of the tapered structures formed after thermal oxidation, we have achieved efficient exosome separation with a purity exceeding 90% while significantly reducing the fabrication costs of the device by circumventing the need for high-precision lithography. Additionally, the incorporation of DEP offers a potential strategy for differentiating exosomes from particles of similar dimensions yet distinct biochemical compositions. This work establishes a robust platform for exosome isolation, thereby facilitating subsequent detection of nucleic acids, proteins, and other exosomal constituents. Moreover, we aim to transfer the DEP simulation outcomes into experimental tests to validate the DEP functionality in our device. Further endeavors will also explore the possibility of topological optimization of the microfluidic design, with aspirations to integrate it with a downstream biosensing apparatus in pursuit of the development of a comprehensive exosome analysis platform.

## Data Availability

The data that support the findings of this study are available from the corresponding author upon reasonable request.

## Supplementary Materials

The following supporting information can be downloaded at: https://www.mdpi.com/article/10.3390/bios14040174/s1, Figure S1: The process flow of the PDMS fabrication; Figure S2: Illustration of forming a tapered structure from the arrays with vertical sidewalls through thermal oxidation; Table S1: The parameters of the silicon-on-insulator wafer; Table S2: The parameters of the deep reactive ion etching (DRIE) process; Video S1: A dynamic video showcasing the movement of particles.

## References

[1] Kalluri R., LeBleu V.S. (2020). The biology, function, and biomedical applications of exosomes. Science.

[2] Pegtel D.M., Gould S.J. (2019). Exosomes. Annu. Rev. Biochem..

[3] Xu R., Rai A., Chen M., Suwakulsiri W., Greening D.W., Simpson R.J. (2018). Extracellular vesicles in cancer—Implications for future improvements in cancer care. Nat. Rev. Clin. Oncol..

[4] Tkach M., Théry C. (2016). Communication by extracellular vesicles: Where we are and where we need to go. Cell.

[5] Valadi H., Ekström K., Bossios A., Sjöstrand M., Lee J.J., Lötvall J.O. (2007). Exosome-mediated transfer of mRNAs and microRNAs is a novel mechanism of genetic exchange between cells. Nat. Cell Biol..

[6] Théry C., Zitvogel L., Amigorena S. (2002). Exosomes: Composition, biogenesis and function. Nat. Rev. Immunol..

[7] Mathivanan S., Fahner C.J., Reid G.E., Simpson R.J. (2012). ExoCarta 2012: Database of exosomal proteins, RNA and lipids. Nucleic Acids Res..

[8] Colombo M., Raposo G., Théry C. (2014). Biogenesis, secretion, and intercellular interactions of exosomes and other extracellular vesicles. Annu. Rev. Cell Dev. Biol..

[9] Théry C., Amigorena S., Raposo G., Clayton A. (2006). Isolation and characterization of exosomes from cell culture supernatants and biological fluids. Curr. Protoc. Cell Biol..

[10] Théry C., Witwer K.W., Aikawa E., Alcaraz M.J., Anderson J.D., Andriantsitohaina R., Antoniou A., Arab T., Archer F., Atkin-Smith G.K. (2018). Minimal information for studies of extracellular vesicles 2018 (MISEV2018): A position statement of the International Society for Extracellular Vesicles and update of the MISEV2014 guidelines. J. Extracell. Vesicles.

[11] Zech D., Rana S., Büchler M.W., Zöller M. (2012). Tumor-exosomes and leukocyte activation: An ambivalent crosstalk. Cell Commun. Signal..

[12] Davies R.T., Kim J., Jang S.C., Choi E.J., Gho Y.S., Park J. (2012). Microfluidic filtration system to isolate extracellular vesicles from blood. Lab Chip.

[13] Guo S.C., Tao S.C., Dawn H. (2018). Microfluidics-based on-a-chip systems for isolating and analysing extracellular vesicles. J. Extracell. Vesicles.

[14] Su W., Li H., Chen W., Qin J. (2019). Microfluidic strategies for label-free exosomes isolation and analysis. TrAC Trends Anal. Chem..

[15] Yuan D., Zhao Q., Yan S., Tang S.Y., Alici G., Zhang J., Li W. (2018). Recent progress of particle migration in viscoelastic fluids. Lab Chip.

[16] Leshansky A.M., Bransky A., Korin N., Dinnar U. (2007). Tunable nonlinear viscoelastic “focusing” in a microfluidic device. Phys. Rev. Lett..

[17] Shao H., Im H., Castro C.M., Breakefield X., Weissleder R., Lee H. (2018). New technologies for analysis of extracellular vesicles. Chem. Rev..

[18] Ding L., Yang X., Gao Z., Effah C.Y., Zhang X., Wu Y., Qu L. (2021). A holistic review of the state-of-the-art microfluidics for exosome separation: An overview of the current status, existing obstacles, and future outlook. Small.

[19] Huang L.R., Cox E.C., Austin R.H., Sturm J.C. (2004). Continuous particle separation through deterministic lateral displacement. Science.

[20] Davis J.A., Inglis D.W., Morton K.J., Lawrence D.A., Huang L.R., Chou S.Y., Sturm J.C., Austin R.H. (2006). Deterministic hydrodynamics: Taking blood apart. Proc. Natl. Acad. Sci. USA.

[21] McGrath J., Jimenez M., Bridle H. (2014). Deterministic lateral displacement for particle separation: A review. Lab Chip.

[22] Voldman J. (2006). Electrical forces for microscale cell manipulation. Annu. Rev. Biomed. Eng..

[23] Shafiee H., Sano M.B., Henslee E.A., Caldwell J.L., Davalos R.V. (2010). Selective isolation of live/dead cells using contactless dielectrophoresis (cDEP). Lab Chip.

[24] Hettiarachchi S., Cha H., Ouyang L., Mudugamuwa A., An H., Kijanka G., Kashaninejad N., Nguyen N.T., Zhang J. (2023). Recent microfluidic advances in submicron to nanoparticle manipulation and separation. Lab Chip.

[25] Wunsch B.H., Smith J.T., Gifford S.M., Wang C., Brink M., Bruce R.L., Austin R.H., Stolovitzky G., Astier Y. (2016). Nanoscale lateral displacement arrays for the separation of exosomes and colloids down to 20 nm. Nat. Nanotechnol..

[26] Davis J.A. (2008). Microfluidic Separation of Blood Components through Deterministic Lateral Displacement. Ph.D. Thesis.

[27] Yang J., Huang Y., Wang X.B., Becker F.F., Gascoyne P.R. (1999). Cell separation on microfabricated electrodes using dielectrophoretic/gravitational field-flow fractionation. Anal. Chem..

[28] Hughes M.P. (2002). Strategies for dielectrophoretic separation in laboratory-on-a-chip systems. Electrophoresis.

[29] Kim D., Sonker M., Ros A. (2018). Dielectrophoresis: From molecular to micrometer-scale analytes. Anal. Chem..

[30] Xing X., Yobas L. (2015). Dielectrophoretic isolation of cells using 3D microelectrodes featuring castellated blocks. Analyst.

[31] di Toma A., Brunetti G., Chiriacò M.S., Ferrara F., Ciminelli C. (2023). A Novel Hybrid Platform for Live/Dead Bacteria Accurate Sorting by On-Chip DEP Device. Int. J. Mol. Sci..

[32] Moore J.H., Varhue W.B., Su Y.H., Linton S.S., Farmehini V., Fox T.E., Matters G.L., Kester M., Swami N.S. (2019). Conductance-based biophysical distinction and microfluidic enrichment of nanovesicles derived from pancreatic tumor cells of varying invasiveness. Anal. Chem..

[33] Van der Pol E., Böing A.N., Harrison P., Sturk A., Nieuwland R. (2012). Classification, functions, and clinical relevance of extracellular vesicles. Pharmacol. Rev..

