# Supplementary material for: A Novel Microfluidic Strategy for Efficient Exosome Separation via Thermally Oxidized Non-Uniform Deterministic Lateral Displacement (DLD) Arrays and Dielectrophoresis (DEP) Synergy
Source: Biosensors (Basel). 2024 Apr 4;14(4):174. doi: 10.3390/bios14040174 (PMC11048442; doi:10.3390/bios14040174)
Supplement: Supplementary file 1 [file biosensors-14-00174-s001.zip › biosensors-2926097-supplementary materials.pdf]

biosensors

Supporting Materials

A Novel Microfluidic Strategy for Efficient Exosome Separation via Thermally Oxidized Non-Uniform Deterministic Lateral Displacement (DLD) Arrays and Dielectrophoresis (DEP) Synergy

Dayin Wang [1,2,3], Shijia Yang [1,2], Ning Wang [1,2,3], Han Guo [1,2], Shilun Feng [1,2], Yuan Luo [1,2,*] and Jianlong Zhao [1,2,3,*]

[1] State Key Laboratory of Transducer Technology, Shanghai Institute of Microsystem and Information Technology, Chinese Academy of Sciences, Shanghai 200050, China; dywang@mail.sim.ac.cn (D.W.)
[2] Center of Materials Science and Optoelectronics Engineering, University of Chinese Academy of Sciences, Beijing 100049, China
[3] School of Information Science and Technology, ShanghaiTech University, Shanghai 201210, China
* Correspondence: yuanluo@mail.sim.ac.cn (Y.L.); jlzhao@mail.sim.ac.cn (J.Z.)

This PDF file includes:

Supplementary Note S1

Supplementary Figures S1 to S3

Supplementary Tables S1 to S2

Descriptions for other Supplementary Materials (movies, audio, etc.) listed below

Other Supplementary Materials for this manuscript include the following:

Supplementary Movie S1

Supplementary Note-S1

We provide a supplementary video from a typical ongoing DLD experiment, which showcases the real-time fluorescent trajectories of 600nm particles within the DLD exosome separation chip. This serves as a supplementary visual aid to the static fluorescence images presented in the main text.

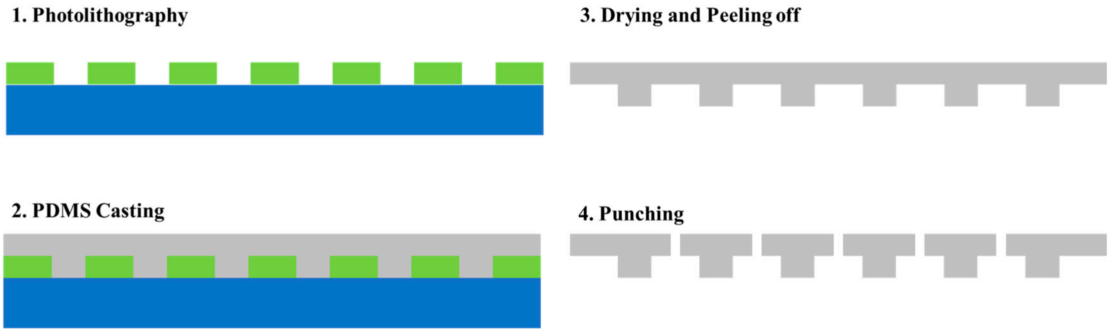

Figure S1. The process flow of the PDMS fabrication.

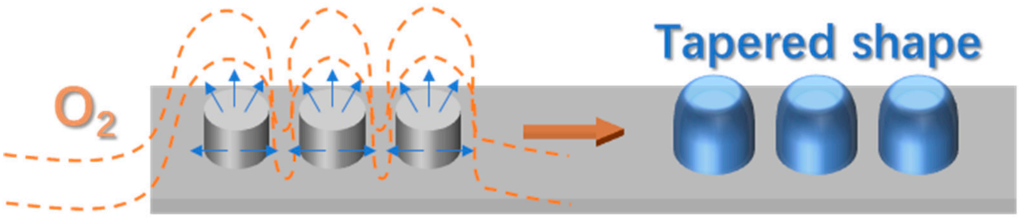

Figure S2. Illustration of forming a tapered structure from the arrays with vertical sidewalls through thermal oxidation.

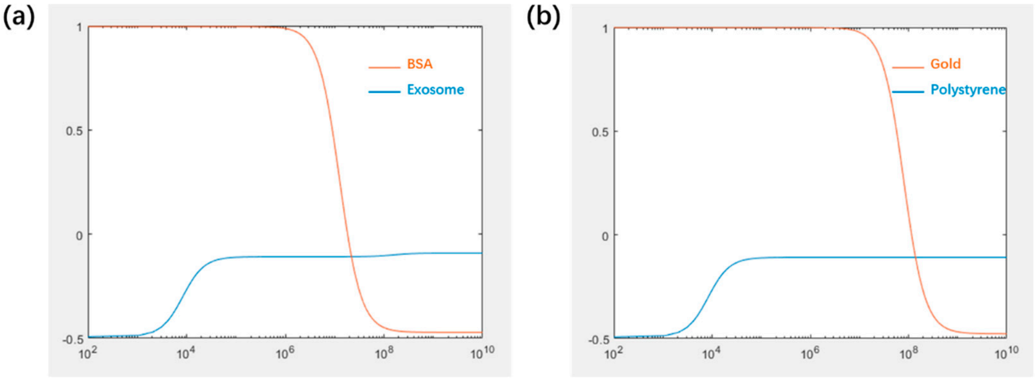

Figure S3. (a)The *Re[f_{CM}]* function for BSA & exosomes; (b) The *Re[f_{CM}]* function for gold nanoparticles & polystyrene nanoparticles.

Table S1. The parameters of the silicon-on insulator wafer.

**Diameter**	4″		
**Device layer**		**Handle wafer**	
**Type/Dopant**	N/As	**Type/Dopant**	N/P
**Resistivity**	0.001~0.005 Ω/cm	**Resistivity**	1~10Ω/cm
**Thickness**	2±0.1μm	**Thickness**	300±15μm
**Orientation**	<100>	**Orientation**	<100>

Table S2. The parameters of Deep Reactive Ion Etching (DRIE) process.

		**Etch**			Passivation		
**Line**	**Gas name**	**Flow (Sccm)**	**Tol(%)**	**Ramp(Sccm/Min)**	Flow(Sccm)	Tol(%)	Ramp(Sccm/Min)
1	C_4F_8	0	50	0.00	85	25	0.00
2	SF_6	130	25	0.00	0	5	0.00
3	O_2	13.0	25	0.00	0.0	5	0.00
4	Ar	0	5	0.00	0	5	0.00
